# Perceived social support and psychological wellbeing among Nepalese adolescents: the mediating role of self-esteem

**DOI:** 10.1186/s40359-020-00409-1

**Published:** 2020-05-01

**Authors:** Anju Poudel, Bishnu Gurung, Gopal Prasad Khanal

**Affiliations:** 1 Tribhuvan University, Institute of Medicine, Pokhara Nursing Campus, Pokhara, Nepal; 2 Pokhara Academy of Health Sciences, Pokhara, Nepal

**Keywords:** Perceived social support, Self-esteem, Psychological wellbeing, Adolescent

## Abstract

**Background:**

Adolescence is characterized by unique, multiple physical, psychological and social development. Understanding the well-being of adolescents and the factors that contribute to it will help towards clarifying and defining ways to better help adolescents prepare for adult life. Therefore, the present study aims to find out the relationship between Perceived Social Support (PSS) and Psychological Well-Being (PWB) among Nepalese adolescence based on mediating role of Self-esteem (SE).

**Methods:**

The study was conducted among 348 adolescents studying in grade 9 and 10 of government secondary level schools of Pokhara Metropolitan city, Nepal. Data were collected through self-administered standard tools-Multidimensional Scale of Perceived Social Support (MSPSS), Rosenberg self-esteem scale (RSES) and General Health Questionnaire (GHQ-12). For statistical analysis descriptive statistics, correlation, regression and mediation analyses were used. The statistical significance of mediating effect of the tested model was examined through a path proposed by Baron and Kenny and Bootstrap method.

**Results:**

Overall, the finding suggests that PSS indirectly affects PWB of adolescents through mediating variable SE. Adolescents who perceive good social support had higher SE, which in turn contributed to their PWB. Furthermore, the study found no significant gender difference for PSS, SE and PWB. Also among various sources of PSS, both boys and girls were more oriented towards family for social support than friends and others.

**Conclusion:**

Adolescents who experience higher social support are likely to have higher SE and are more likely to have better PWB. The findings of the study will be useful to the parents, teachers, counselors, psychologist and researchers to develop strategies to enhance adolescent’s mental health.

## Background

Adolescence is a period of transition from childhood into adulthood. It is marked by rapid physical and psychological transformation. Compared to children, adolescents encounter many stressful situations, potentially threatening or challenging social experiences. Major stressors are related to interpersonal relationships (eg. conflicts with parents, siblings and peers) [1–3], financial pressure and school performance (eg. academic failure, low achievement) [2, 4].

During this period, adolescence changes in the way they interact with their family and peers. However, individual differences are noted on how they perceive that support from each source. The youngsters begin to demand greater autonomy and an altered relationship with parents. At the same time peers plays significant role in adolescents’ emergence from family towards independence [5, 6]. However parents still remain strong socializing agents throughout the period and this positive relationship act to enhance self-esteem among adolescents [7, 8]. Extensive number of literatures have consistently shown that youth derive social support from a number of sources (e.g., parent/family, peers/classmates, relatives and teachers), and social support from each source is associated with beneficial outcomes [9]. Social support can be emotional, instrumental, financial or informational [10].

Several studies have provided strong evidence of the relationship between social support and psychological well-being (PWB). It helps an individual to reduce the amount of stress experienced as well as act as a buffer for individual facing stressful life situations [11]. Social support could act as protective factor and plays an important role in PWB among adolescence [11, 12]. As per Wethington and Kessler, perceived social support (PSS) is more important than received social support [13]. They also provide capacity needed for adolescent for PWB in adulthood [14, 15]. Perceptions of positive support from family members have been linked with increase in indicators of wellness such as life satisfaction [16]. Suldo and Schaffer analyzed mental well-being among youth and found that peer support correlate inversely with other indicators of internalizing psychopathology in adolescents and co-occur with psychological wellness among adolescents [17].

Among risk and protective factors associated with PWB during adolescence, self-esteem (SE) and relationship with parents plays a relevant role. SE is based on the conception of one’s own worthiness, which is determined not only by self-perceptions but also by interpretations of feedback from significant others [18]. A high level of SE is commonly associated with PWB [19] and happiness [20]. Studies have demonstrated statistically significant positive relationship between different agents of PSS and SE among adolescents [21]. Similarly support from friends also foster SE in adolescent especially in the adolescence years [22]. PSS and SE partially mediate the relationship between paternal and maternal attachment and life satisfaction [16]. Parental support could promote SE in children and reduce psychological distress by offering their support throughout this developmental phase [23]. In addition, adolescence who receive high parental support has better SE than those who receive low parental support [24, 25]. Furthermore, it has been reported that SE is an important mediator between social support and internalizing symptoms in adolescents [26]. A study conducted among Chinese adolescents reported that for early adolescents, global self-esteem mediated relations of parent and teacher support with school well-being; whereas for middle adolescents, global self-esteem mediated relations of friend and teacher support with school well-being [14].

Social support is generally perceived differently among male and female. Studies have reported higher levels of psychological distress among girls than boys [23, 27]. In regard to the sources of support, girls perceive more social support than boys did [28]. Girls find more support from close friends than any other sources, whereas boys perceive less from all [29, 30]. Female adolescents as compared to male adolescents are more oriented toward peers for social support and are also more satisfied with the support gained from their peers [31]. However, no gender differences in SE and PSS were reported in the study conducted among the Malaysian adolescents [32]. Based on the literature review it can be concluded that much research has been done in relation to gender differences in PSS, SE and PWB but results are inconclusive.

Similarly, in recent years, there has been a dramatic increase in the scientific study of well-being and positive aspects of mental health. Numerous studies investigated the relationships between PSS, SE, and different components of wellbeing such as subjective wellbeing, life satisfaction, school wellbeing, happiness [14, 16] but few researches up to date have examined the mediating role of self-esteem on relationships between PSS and PWB among adolescents. Furthermore, most of the studies which have highlighted the significant relationship between these variables are from other cultural background and it is not clearly demonstrated whether the findings can be generalized to our culture as well. As per the researcher’s knowledge none of the studies from Nepal has examined this association. In addition, it is well known fact that being psychologically healthy is important for adolescent development in general, but less is known about the predictors of PWB and the mediating role of SE on relationship between PSS and PWB. So, the current study aims to contribute to the literature by further delineating the complex relationships between the aforementioned variables in adolescent sample from developing county like Nepal. The study will also expand the scope of mental health by identifying the predictors of psychological wellbeing as they could inform preventive interventions. An understanding of associations between PSS, SE and PWB will provide a more complete picture of psychological functioning and its buffers. Such information can be used to help inform prevention and intervention efforts regarding wellness promotion in adolescents.

Therefore, the study is planned with the aim to answer three research questions: 1) Is there any gender differences in various sources of PSS, SE and PWB among adolescents? 2) What is the relationship between PSS, SE and PWB among adolescents 3) How do SE mediates the relationship between PSS and PWB among adolescents?

## Methods

### Study design

A descriptive cross section research design was used.

### Participants and procedure

A total of 348 undergraduate students of grade 9 and 10 participated in the study, of which 146 (42%) were male and 202(58%) were female. The mean age of the participants was 15.30 ± 1.16, ranging from 13 to 20 years.

Multistage cluster sampling technique was used. To ascertain similar environment for boys and girls, only co-educational schools were chosen. At first, the different wards of Pokhara Metropolitan city were regarded as clusters. In the second stage, simple random sampling technique was applied to select 5 wards among 33 wards. The list of all government secondary level schools of selected wards was enumerated. Then, from among the list of government schools, simple random sampling technique was applied to choose one school from each ward. The final sample consisted of 348 undergraduate students of grade 9 and 10 from the selected schools.

The study was approved by the Institutional Review Board of Institute of Medicine, Tribhuvan University, Nepal. Administrative approval was obtained from the authorities of selected schools. With the help of the staff of the schools all the students willing to participate in the study were kept in one hall. Informed consent was obtained from each student prior to data collection. Parents’ informed assent was also obtained before participation. Students were also informed about the purpose of the study, their voluntariness in participation and no any foreseeable risk and harm in the study.

### Measures

#### Demographic questionnaire

Variables assessed in the demographics questionnaire included participants’ age, gender and grade.

#### Perceive social support (PSS)

PSS was assessed by using Multidimensional Scale of Perceived Social Support (MSPSS) [33]. The 12 items self-report measure provides a subjective assessment of social support from family, friends, and others. Each item can be scored using a 7-point Likert scale (1 = very strongly disagree; 7 = very strongly agree). Subscale scores can be calculated by summing related responses, with higher scores indicating a higher degree of PSS from that particular source. The MSPSS has shown high internal reliability (Cronbach’s alpha = .87, .85, and .91 respectively for the Family, Friends and Significant others subscales) [33]. Cronbach’s alpha coefficients for the present sample were 0.75 for the family subscale, 0.80 for the friends subscale, 0.77 for the others subscale, and 0.82 for the full scale.

#### Self-esteem (SE)

The Rosenberg Self-esteem scale (RSES) is a 10-item measure that assesses an individual’s overall evaluation of his or her worth or value [34]. Globally, RSES is the most widely used scale to measure SE among adolescents and has been shown to have good reliability. The items are rated using a 4-point likert scale (1 = strongly disagree; 4 = strongly agree). The scoring is obtained by doing the sum of scores according to the ratings assigned to all the items after reverse scoring the negatively worded items and score ranges from 4 to 40. The internal consistency reliability of tool ranges from .85 to .88 for college samples [34], whereas in the current study it was computed as 0.71.

#### Psychological well-being (PWB)

PWB was measured by using the 12-item version of the General Health Questionnaire - GHQ-12 [35]. It was devised as a short version of the original 60-item questionnaire. The GHQ-12 is a reliable and convenient self-rated questionnaire with adequate sensitivity and specificity, and has been frequently used to assess the PWB [36, 37]. Likert-type scoring (0–1–2-3) is applied. Sum scores range from 0 to 36, with lower scores indicating better PWB. The cronbach’s alpha coefficient for the GHQ-12 in the current study was 0.90.

Data were collected by using Nepali version, pretested self-administered questionnaire. The tool was first forward translated (from English to Nepali) then backward translated (Nepali to English). Finally, the differences between original and back translated texts were resolved through discussion between translators for ensuring semantic equivalence.

### Statistical analysis

Statistical Package for Social Sciences (Version 20; IBM Corporation, Armonk, NY, USA) was used for data analysis. Normality was verified using Shapiro–Wilk test, and the data were found to be normally distributed (*p* > 0.05). The data was analyzed by using descriptive statistics such as frequency, percentage, mean and standard deviation. For inferential analysis t-test was used. In all the inferential statistical procedures, *p* value of 0.05 or less (*p* ≤ 0.05) was considered statistically significant. The Pearson’s correlation coefficient (*r*) was calculated to assess the relationship among PSS, SE and PWB variables.

To examine the mediating effect, the path proposed by Baron and Kenny was employed [38]. It was hypothesized that SE mediates the relationship between PSS and PWB in adolescents. In the first equation, the mediator (SE) was regressed on the independent/predictor variable (PSS). In the second equation, the dependent variable (PWB) was regressed on the independent variable (PSS). In the third equation, the dependent variable (PWB) was regressed on the mediator (SE) and the independent variable (PSS). In addition to these 3 regression equations verifying the relationship among the mediation models, the bootstrapping method was used to examine the reliability of mediating effects. The PROCESS macro for SPSS (version 22) was used to perform mediation analysis [39]. In order to test the significance of indirect effects, we used five thousand 95% bootstrap confidence intervals (CI). An indirect effect was considered to be significant at the 0.05 level if the 95% CI did not include zero.

## Results

The mean and standard deviation score of PSS, its subscale, SE and PWB were identified and independent sample t-test was used to compare between boys and girls (Table 1). None of the variables were found to have statistically significant differences by gender. Furthermore, among various sources of PSS, both boys and girls were more oriented toward family for social support than friends and others.

**Table 1:** Gender differences on PSS, SE and PWB

**Variables**	**Boys (*n*** **= 146) Mean ± SD**	**Girls (*n*** **= 202) Mean ± SD**	**t**	***p*value**
**Total PSS**^**a**^	62.47 ± 13.05	61.87 ± 16.80	0.37	.719
**PSS from family**^**a**^	23.21 ± 5.13	22.32 ± 6.44	1.43	0.15
**PSS from friend**^**a**^	19.82 ± 5.35	20.00 ± 5.64	−0.30	0.76
**PSS from significant other**^**a**^	19.45 ± 4.91	19.55 ± 6.50	−0.17	0.85
**SE**^**b**^	28.49 ± 3.20	27.92 ± 2.98	1.71	0.87
**PWB**^**c**^	9.77 ± 6.03	9.89 ± 6.63	−0.16	0.87

^a^Higher score indicates higher PSS; Total PSS min.: 7, max.: 84; PSS from family, friend and other min.: 4, max.: 28

^b^Higher score indicates higher SE; min.: 4, max.: 40

^c^Higher score indicates lower PWB; min.: 0, max.: 36

*p* significant at ≤0.05 level of significance

### Correlational analyses

Pearson product-moment correlations among all continuous variables included in analyses are presented in Table 2. Total PSS was significantly correlated with its subscale, SE and PWB. Furthermore, the subscales of PSS (i.e., Family, Friend and Significant other scales) were all significantly correlated with one another. However, PSS from family and friend were not significantly correlated with SE. This means, as total PSS and PSS from significant other increases, SE also increases. Furthermore, SE also showed significant relationship with PWB. Higher the SE, total PSS, PSS from family and significant other, better the PWB.

**Table 2:** Correlations among PSS, SE and PWB

**Variables**	**Total PSS**	**PSS form family**	**PSS from friend**	**PSS form significant other**	**SE**	**PWB**
**Total PSS**	1	0.90^b^	0.86^b^	0.88^b^	0.12^a^	− 0.13^a^
**PSS form family**		1	0.67^b^	0.70^b^	0.09	−0.11^a^
**PSS from friend**			1	0.62^b^	0.10	0.09
**PSS form significant other**				1	0.13^a^	−0.15^b^
**SE**					1	−0.37^b^
**PWB**						1

^a^value of correlation significance at 0.05 level

^b^value of correlation significance at 0.01 level

### Mediating effect test

**Fig. 1:**
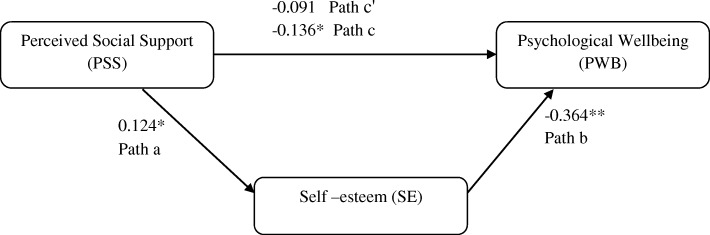
Mediation model showing role of Self-esteem on relationship between Perceived Social Support and Psychological Well-being. Values shown are standardized coefficients *p < 0.05, **p < 0.01

The results of the mediation analysis are shown in Fig. 1, Table 3. The PSS was significantly associated with SE (β = 0.124, *p* < 0.05). SE was also found to have significant association with PWB (β = − 0.364,*P* < 0.05). Moreover, the total effect of PSS on PWB was significant (β = − 0.136, *p* < 0.05). When SE was controlled, the previously significant relationship between the predictor PSS and outcome PWB (direct effect) became non-significant (β = − 0.091, *p* = 0.06). The bootstrapping confidence intervals (CI) revealed that the indirect effect of PSS on PWB via SE did not include zero (CI = − 0.087, − 0.007; indirect effect = − 0.045), indicating that the model was reliable and relationship between PSS and PWB was fully mediated by SE. Approximately 15% of the variance in PWB was accounted by the predictors (R^2^ = 0.149).

**Table 3:** Mediating Effect of Self-esteem between Perceived Social Support and Psychological Wellbeing (*n* = 348)

**Testing steps in mediation model**	**B**	**SE**	**t**	**95% CI** **LLCI, ULCI**	**β**
**Testing step 1 (Path a)**					
Predictor (PSS) to Mediator (SE)	0.025	0.010	2.337	0.004, 0.046	0.124*
**Testing step 2 (Path c)**					
Predictor (PSS) to Outcome (PWB)	−0.056	0.022	−2.567	− 0.100,-0.013	− 0.136*
**Testing step 3 (Path c’ and Path b)**					
Predictor (PSS),	− 0.038	0.020	−1.824	− 0.079,0.003	− 0.091
Mediator (SE)	− 0.753	0.103	−7.272	− 0.957,-0.549	− 0.364**
to Outcome (PWB)					

* *p <* 0.05; ** *p <* 0.01, B = Un-standardized Regression Coefficient; *SE* Standard Error, *LLCI* Lower Level Confidence Interval, *ULCI* Upper Level Confidence Interval; β = Standardized Regression Coefficient

## Discussion

The current study was designed to examine the role of SE on relationship between PSS and PWB in a sample of Nepalese adolescents. Correlation analysis showed that PSS, SE and PWB had significant relationship with each other. These results are consistent with previous studies that reported the relationship between PSS and SE [14, 16, 21, 32, 40–43], PSS and different aspects of wellbeing [12, 14–16, 43–45], SE and wellbeing as well as happiness [14, 16, 20, 43, 46]. Moreover, the most important finding of this study is that SE fully mediated the relationship between PSS and PWB suggesting adolescents with high levels of social support are likely to have higher SE which in turn enhances their PWB.

Various studies have identified SE as independent, dependent as well as mediating variable. Consistent to present study finding the higher level of PSS has been shown to predict higher SE in number of previous studies [43, 47–49]. The study showed that PSS plays a significant role in adolescent’s SE formation. The attitudes of goodwill, nurturance and attachment foster the kind of environment to the adolescents which contribute to promote high SE. This finding recommend the need of provision of socially supportive environment to adolescents in the home and school settings which helps to develop positive regard for themselves in the form of high SE. In agreement with earlier findings [43, 47–49], the current study also found SE as the significant predictor of PWB. This finding has been supported by the study conducted in New Zealand which revealed that adolescents with low SE had poorer mental and physical health and low SE predicted negative outcomes in their adulthood [46]. In addition, study among Scottish adolescents also suggested SE as the important predictor of well-being [50]. Furthermore, higher SE has been shown to act as a protective factor against mental health problems in young adults and adolescents [51, 52].

In spite of the previous studies pointing out social support as the significant predictor of wellbeing [43, 45, 47–49, 53], in the present study the direct effect of PSS on PWB was insignificant. However, the relationship was established with the help of mediating variable SE. PSS explained 15% of variance in PWB with mediating variable SE. In line with this other studies have also indicated that PSS relate to different aspects of wellbeing through adolescents perception of self-worth that i.e. SE [14, 47–49, 54, 55]. Yarcheski et al. study reported that self-esteem mediated the relationship between social support and general well-being in American early adolescents [47]. Similarly, among Chinese adolescents global self-esteem partially mediated the influence of social support on life satisfaction and positive affect [48]. The study identified that both individual and environmental factors together affects the individual’s wellbeing status. Furthermore, it suggests that adequate social support improves adolescents SE, thus impacting their PWB.

In this study, there was no significant gender difference for PSS, SE and PWB. The result is identical to the study conducted in Malaysia which found no significant differences for PSS and SE among male and female students [32]. Study conducted among Turkish adolescents also found no gender differences in SE [21]. Similarly, study conducted among university students in India found no gender differences for PSS from family but a significant difference was found out for the PSS from friends [44]. However other study done among adolescents indicated gender differences, boys had higher SE [23, 42, 56] and PWB [57] than girls.

Social support from family, peers, and significant others has been recognized as a protective factor for adolescents. The present study found that among various sources of PSS, both boys and girls were more oriented towards family for social support than friends and others. This might be due to the cultural factor wherein continued parental care and family involvement even at this stage made the adolescent perceive to have better support from family than others. Some research has indicated that adolescents turn more towards peers for social support from middle childhood [28, 58, 59]. However, other study have pointed that parents still continue to provide the secure base from which adolescents explore other relationships [8]. Similarly, inconsistent to our finding a study done in Malaysia also documented peer support as the highest form of social support among adolescents [32]. Furthermore, Colarossi also suggested that female as compared to male adolescents are more oriented toward peers for social support and are also more satisfied with the support gained from their peers [31].

Research about social support carried out in different contexts and cultures have demonstrated that there is a strong relationship between social support system and well-being. The current study replicates the findings of other studies, however on some aspects it differed from previous studies. As discussed earlier, in the present study the direct effect of PSS on PWB was insignificant. In addition, though SE buffered the relationship between PSS and PWB, the effect was lower than that found in most of the other studies [14, 47]. This incoherent finding might be due to differences in individual characteristics and cultural background of adolescents of different samples. Furthermore, considerable changes have been found on social relationship of adolescents over the past years. They are having greater autonomy from parents and relatives and spend less time with them. They try to establish their own identity which is separate from their parents. In addition, adolescents are becoming more individualistic and copying some values and practices from individualist culture [60]. The importance and need of social support now might have been reduced by engagement of adolescents in some other work such as social media which offer new opportunities for leisure, shopping, and staying in touch with others, as well as broader access to information and support. By taking importance to these results, it can be said that the relation between PSS and PWB is more complex than it is thought. Therefore, more research will in fact be necessary to refine and further elaborate our findings. Furthermore, additional research is likely needed to identify other mediators so that the theory regarding the relationship between PSS and PWB can be further developed.

## Conclusion and implications

Overall, the result of current study revealed the importance of PSS and SE for the psychological development of adolescents. The findings suggest that for Nepalese adolescents, PSS indirectly affects PWB through their SE. PSS was found as a significant predictor of SE and SE was also a significant predictor of PWB. Adolescents who perceive good social support had higher SE, which in turn contributed to their PWB. Furthermore, the study found no significant gender difference for PSS, SE and PWB. Also among various sources of PSS, both boys and girls were more oriented towards family for social support than friends and others.

The current findings have several important implications. The findings demonstrated that PSS influences adolescents’ PWB via SE, which indicates that higher level of SE is pivotal for individual’s sense of wellbeing. Improving social support by means of various sources may alleviate distress and foster SE. The findings could help educators, counselors, and psychologist to design and develop proper intervention program to reduce psychological problems among adolescents. Parents and educators should be aware of the importance of enhancing SE among adolescents so that psychological problems might be under control.

### Limitations

The finding of this study has to be considered with reference to its limitations. Nepal Demographic Health Survey 2016 reports that only 82% of girls and 81% of boys aged 11 to 15 attend schools in province 4 which includes Pokhara Metropolitan City [61]. Since the study enrolled the adolescents from schools only, the children who are drop out of schools and are more likely to have lower social support and SE were missed. This absence limits the generalizability of our findings to adolescents who are left out of school. The other limitation is that the study findings relied on the response of students from self-administered questionnaire which may be influenced by recall bias, under-reporting of information and subjective to students’ response. Similarly, the cross-sectional nature of the study prevents establishing a causal relationship between the variables. To solve this problem and identify the causal relationships, longitudinal and experimental studies are needed. Furthermore, the study enrolled students from public schools of Pokhara Metropolitian city only so cautions should be taken while generalizing these findings to other parts of Nepal and to those attending private schools. The other limitation is that the mediating role of SE has been demonstrated in this study, but other mediators need to be identified and tested. Despite these limitations, there are important contributions in this study. The current study provides an empirical framework for the researchers through testing the mediating effects of SE between PSS and PWB in a sample of Nepalese adolescents.

## Data Availability

The datasets generated and analyzed during the current study will be available from the author upon reasonable request. Regarding different tools used in this study, it is a copyright tool. The translated Nepali version of this tool are however available from the corresponding author upon request and with permission obtained from copyright owner of the tool.

## Abbreviations

PSS: Perceived Social Support
SE: Self-esteem
PWB: Psychological Well-Being
MSPSS: Multidimensional Scale of Perceived Social Support
RSES: Rosenberg self-esteem scale
GHQ-12: General Health Questionnaire 12
